# Supplementary material for: MetaRibo-Seq measures translation in microbiomes
Source: Nat Commun. 2020 Jun 29;11:3268. doi: 10.1038/s41467-020-17081-z (PMC7324362; doi:10.1038/s41467-020-17081-z)
Supplement: Supplementary file 10 — Supplementary Data 7 [file 41467_2020_17081_MOESM10_ESM.zip › File2/Confidence_High_Taxonomy/108018_out.krona.html]

Javascript must be enabled to view this page.

members
magnitude
magnitudeUnassigned
count
unassigned
taxon
rank

108018\_out

21

2
21
superkingdom

phylum
21
1239

186801
21
class


SRS012273\_contig\_number\_contig-100\_4002.219383SRS012969\_contig\_number\_contig-100\_8899.94491SRS013687\_contig\_number\_contig-100\_3462.187297SRS014235\_contig\_number\_contig-100\_3549.247138SRS019068\_contig\_number\_13438SRS020328\_contig\_number\_36251SRS042966\_contig\_number\_19939SRS049773\_contig\_number\_27817SRS049959\_contig\_number\_48080SRS053356\_contig\_number\_contig-100\_10508.179162SRS065504\_contig\_number\_14804SRS1041039\_contig\_number\_1447SRS1041137\_contig\_number\_contig-100\_21931.21932SRS104400\_contig\_number\_49302SRS142599\_contig\_number\_22840SRS143895\_contig\_number\_38812SRS144183\_contig\_number\_34115SRS146812\_contig\_number\_6262SRS147557\_contig\_number\_29736SRS148196\_contig\_number\_9041
20
species
2044939

186802
order
1

31979
family
1

1485
1
genus

1262845
species
1

SRS011134\_contig\_number\_8056
